# Advancing child health and educational equity during the COVID-19 pandemic through science and advocacy

**DOI:** 10.1186/s13584-021-00512-7

**Published:** 2022-01-07

**Authors:** Rachel Gur-Arie, Sara Johnson, Megan Collins

**Affiliations:** 1 Johns Hopkins University Berman Institute of Bioethics, Baltimore, MD USA; 2 Department of Pediatrics, Johns Hopkins University School of Medicine, Baltimore, MD USA; 3 Departments of Mental Health and Population, Family and Reproductive Health, Johns Hopkins Bloomberg School of Public Health, Baltimore, MD USA; 4 Wilmer Eye Institute, Johns Hopkins School of Medicine, 600 North Wolfe Street, Wilmer 233, Baltimore, MD 21231 USA

**Keywords:** Educational achievement, COVID-19, Child health, Bioethics, Health disparity, School health services

## Abstract

The COVID-19 pandemic has underscored the changing role of scientists, clinicians, ethicists, and educators in advocacy as they rapidly translate their findings to inform practice and policy. Critical efforts have been directed towards understanding child well-being, especially with pandemic-related educational disruptions. While school closures were part of early widespread public health measures to curb the spread of COVID-19, they have not been without consequences for all children, and especially for children from disadvantaged backgrounds. In a recent *Isr J Health Policy Res* perspective, Paltiel and colleagues demonstrate the integral role of academic activism to promote child well-being during the pandemic by highlighting work of the multidisciplinary academic group on children and coronavirus (MACC). In this commentary, we explore parallels to MACC’s work in an international context by describing the efforts of a multidisciplinary team at Johns Hopkins University in Baltimore, Maryland, United States, to aggregate data, conduct analyses, and offer training tools intended to minimize health and educational inequities for children throughout the COVID-19 pandemic. As both MACC and our work collectively demonstrates, multidisciplinary partnerships and public-facing data-driven initiatives are crucial to advocating for children's equitable access to quality health and education. This will likely not be the last pandemic that children experience in their lifetime. As such, efforts should be made to apply the lessons learned during the current pandemic to strengthen multidisciplinary academic-public partnerships which will continue to play a critical role in the future.

The COVID-19 pandemic has catalyzed or accelerated countless changes in the conduct and dissemination of biomedical research, such as unprecedented collaboration on COVID-19 vaccine candidates and the proliferation of preprint manuscripts for the rapid release of preliminary scientific discoveries [1, 2]. The pandemic has also underscored the changing role of scientists in translating their scientific findings to practice and policy [3]. Nowhere has this been more evident than in ongoing decisions about preventing viral transmission in schools during the pandemic. Children have been among the most impacted by large-scale public health interventions employed to reduce the risk of COVID-19 transmission, including school closures, virtual learning, and social distancing. Moreover, learning disruptions have exacerbated pre-existing equity and achievement gaps [4].

The urgency and scope of the COVID-19 pandemic have nudged researchers out of the relative safety of the academy, and they are increasingly wading in to provide tools and resources that drive policy and practice in real-time. In the recent issue of the *Israel Journal of Health Policy Research*, Paltiel et al. provided an important case study of how the *multidisciplinary academic group on children and coronavirus* (MACC) offered epidemiological, clinical, and health policy advice to decision-makers regarding choices impacting children throughout the pandemic in Israel [5]. The MACC’s work included evidence-based guidance regarding school closures as a pandemic control intervention and suggestions for safe school reopening. Tensions that arose between the MACC and government decision-makers revealed the challenges surrounding balancing dynamic scientific evidence with political influences when making decisions for a vulnerable population, like children during a pandemic emergency. Paltiel et al. recognized the value of leaning into an advocacy role by providing the “rigor of their scientific work and perceived objectivity” to inform government and public health decision-making processes. Central to MACC’s work exploring the role of COVID-19 transmission in schools was the recognition that education is a social determinant of health. There is substantial research evidence to demonstrate the critical role of the educational system in lessening achievement and health inequities; MACC recognized that pandemic-related school disruptions could risk widening achievement gaps and exacerbating pre-existing inequities. The grass-roots approach that MACC took to mobilize forces important to decisions about school reopening is an example for academics worldwide interested in advocating for the well-being of children during the current pandemic and beyond.

As a multidisciplinary team at Johns Hopkins University in Baltimore, Maryland, we had similar experiences to those described by Paltiel et al. Here we discuss our efforts to provide technical assistance, aggregate data, conduct analyses, and offer training tools intended to minimize health and educational inequities for children throughout the COVID-19 pandemic. This commentary is intended to expand upon the lessons from MACC and argue for the urgency of continuing this work, even after the COVID-19 pandemic subsides.

## Academic advocacy through technical assistance and educational outreach

At the onset of the pandemic, as schools began to close, the critical links between health and education became even more evident. Together, the Johns Hopkins Consortium for School-Based Health Solutions, the nexus of school-based health research and practice at Johns Hopkins University, and the eSchool + Initiative, a collaboration between faculty from the Schools of Medicine, Education, Public Health and Berman Institute of Bioethics, have worked to bring attention to the disproportionate impact of the pandemic on children who were vulnerable to poor health and academic outcomes before the pandemic. We have focused on: (1) providing evidence-based COVID-19 transmission mitigation technical assistance to schools and districts, and (2) aggregating, distilling, and disseminating data needed to monitor equity gaps in learning and health around the United States (US) and globally.

Like the MACC’s work in Israel, we brought together academic experts from school health, education, public health, ethics and equity, and health policy. We contributed to multiple ongoing efforts in parallel, such as offering consultations to policy organizations and technical assistance to local schools, districts, and health departments, and disseminating our work through webinars and web-based platforms that were freely available to a local, national, and international audience.

Early in the pandemic, evidence-based operational decision support for schools and districts was in high demand. Educators struggled to devise public health mitigation plans that would allow them to provide in-person instruction safely in the absence of national or state guidelines. Was SARS-CoV-2 airborne? Were plastic barriers needed between students in the classroom? To what extent did students need to be physically distanced in classrooms or on school buses? How many times per day did classroom surfaces need to be cleaned? We worked directly with school districts in several US states to provide ongoing technical assistance and decision support informed by our evolving understanding of viral transmission and in-school transmission risk. Some of the questions that have come up in discussion include teacher vaccine mandates, testing protocols, and the attendant equity issues related to any school or district-wide policy decisions.

To expand access to this information beyond the districts with which we worked directly, we designed a series of free, web-based, school-reopening modules for school and district administrators [6]. The modules addressed topics ranging from mitigation strategies like face coverings, cohorting (i.e., minimizing mixing between separate groups of students), ventilation, COVID-19 transmission, viral screening and testing, COVID-19 case monitoring, and communication with the school community.

## Applying an equity lens to track US policy decisions about K-12 school operations during the pandemic

School closures and ongoing educational disruptions have not been without short and long-term consequences for students—even more so for those who may have been struggling before the pandemic began [4, 7]. As MACC recognized in their advocacy efforts in Israel, schools address more than just the educational needs of students; they are also critical links to food assistance, social support, developmental supports, screening services, and physical and mental healthcare [8, 9]. Early in the pandemic, while substantial attention was initially being directed to supporting remote learning in the US, considerably less effort was invested into accounting for the loss of additional supports that schools routinely provided, especially for students of systemic disadvantage [10, 11]. To address this gap, in parallel to our work providing technical assistance and developing COVID-19 educational modules, we also created guidance documents designed to assist policymakers in critical decision-making for Kindergarten (K)–Grade 12 school closings and reopening, employing an equity lens to focus on the disproportionate impact that school disruptions could pose to the most vulnerable children.

To assist policymakers, educators, and public health professionals in monitoring the changing landscape of school COVID-19 policies and practices, we also embarked on several data-driven initiatives that harnessed publicly available data about K—Grade 12 school responses during the pandemic. Our earliest data project, the 2020–2021 School Reopening Tracker, monitored state department of education reopening plans for all 50 states, the District of Columbia, the Bureau of Indian Education, and major US territories [12]. We displayed these data in real-time on a publicly available website and tracked how state plans addressed both operational and equity considerations over time [12]. At the launch of the tracker, in July 2020, there was less attention to equity considerations in school reopening plans, with significant gaps in support systems needed for children of systemic disadvantage. By using a public-facing data initiative, we hoped that notable failures to address equity considerations would serve as a call to action for expanding state resources for those who were suffering the most. And as time passed, more states began updating their reopening plans to include some of these critical elements. In a time-trend analysis between the July 2020 launch of our tracker and December 2020, there was a measurable increase in plans adding details about equity considerations. While we cannot establish a causal relationship between our tracker and the updates to state reopening plans, it is worth noting that several media outlets covered stories about the 2020–2021 School Reopening Tracker using the tracker as a point of comparison between their plans and others, sometimes to highlight how they compared favorably to surrounding states [13, 14].

When Moderna and Pfizer COVID-19 vaccines received emergency use authorization (EUA) for adults from the US Food & Drug Administration (FDA) in late 2020, we recognized how pivotal teachers’ COVID-19 vaccine access would be to schools returning fully to in-person learning. Accordingly, we launched the Teacher & School Staff COVID-19 Vaccination Dashboard, which collected data from state vaccination plans and tracked changes in teacher eligibility for COVID-19 vaccination across the US over time [15]. Both the School Reopening Tracker and the Teacher & School Staff Vaccination COVID-19 Dashboard were updated regularly to reflect evolving policies at the state level. In late 2021, we launched the 2021–2022 National and Index School District Mask and COVID-19 Vaccine Policy Tracker, capturing mask and vaccine policy data from US states and an index sample of school districts [16]. This tracker includes comparative analyses that highlight differences in mask and vaccine policies at the state and district level, as well as variation among the highest and lowest poverty districts and largest districts. It is designed to both bring public attention to the accumulating body of evidence about variation in school COVID-19 policies and to examine which policies risk worsening existing inequities in access to educational resources.

## Monitoring school reopening decisions globally

In addition to our work in the US, we partnered with World Bank and UNICEF to collaborate on the COVID-19 Global Education Recovery Tracker (GERT) in late 2020. The GERT captures publicly available data on the state of education across all grade levels, including in-person academic supports, remote learning modalities, and teacher prioritization for COVID-19 vaccines from more than 200 countries and territories worldwide [17]. The data is displayed on a publicly available dashboard. It is freely accessible and intended to support educators, policymakers, and researchers as we pivot from the emergency-oriented educational response of 2020 to remediation and recovery efforts in 2021 and beyond. One such example of our work’s impact relates to the UNESCO-World Bank Group-UNICEF ‘Mission: Recovering Education in 2021’ report, which recognized that simply reopening schools to normal pre-pandemic operations would not be enough; in reopening schools, education systems also needed to account for the additional services and supports students would need to meet their “learning, health, psychosocial wellbeing, and other needs [18].” The report cited GERT as a key data source to monitor progress towards this target.

## Lessons learned from academic-public advocacy partnerships during the pandemic

During the COVID-19 pandemic, academic-public partnerships have proven essential in advocating for child well-being, employing both educational outreach and data-driven initiatives with local, national, and global stakeholders. We have utilized multiple avenues to disseminate findings promptly and to assist policymakers and education stakeholders in real-time. These have included opinion editorials (“op-eds”) in local and national newspapers, webinars, electronic newsletters, podcasts, blog posts, and peer-reviewed articles [19–25].

Our work brought attention to data and policy decisions around K-12 education during that pandemic that could impact the well-being of children, especially the most disadvantaged; however like MACC, we were unable to offer stakeholders a budget model for how to mitigate some of these inequities. This is especially relevant in the US where there are more than 14,000 public school districts. Education-related policy and funding decisions in the US are largely made at the state and district level. This has resulted in significant variation across the country in policy and funding decisions regarding child well-being throughout the COVID-19 pandemic. The discordance in policy decisions across the US, oftentimes in combination with markedly different local and state budget resources, poses an additional equity challenge; not all schools or school districts can implement public health mitigation in the same manner, and this may impact the safety of a school staying open, as seen recently with ventilation systems in New York City Public Schools [26], or even parent willingness to send their children back to the classroom [27, 28].

While MACC reported limited involvement of education experts weighing into decisions about school reopening in Israel, there was a proliferation of US-based trackers monitoring school reopening, and academic experts from across disciplines joining public discussions about the best path forward in education [5, 7, 29–32]. In this space, our lens on equity and engagement through education and technical assistance provided a unique and valuable way to contribute meaningfully in real-time, while also creating data resources that facilitate monitoring of child well-being into the future.

## Conclusions

Like MACC’s experience in Israel, building partnerships, aggregating and displaying data in real-time, and disseminating findings through web-based platforms was a different role for our US-based multidisciplinary research team of clinicians, educators, bioethicists, and public health professionals. The quickly-moving COVID-19 pandemic necessitated a new system—beyond traditional peer review—to rapidly collect and disseminate evidence to promote the needs of vulnerable children and families during the pandemic response and recovery efforts. These multidisciplinary partnerships and public-facing data-driven initiatives are critical to sustaining and continuing to advocate for children's equitable access to quality health and education. The advocacy-focused approach adopted by Paltiel et al. and our data aggregation approach can serve as models for how academics can advocate for, and ultimately improve, child well-being during times of uncertainty and evolving scientific evidence. A critical reality raised by Paltiel and our team is that this will likely not be the last pandemic our children experience in their lifetime and there is a role for these new partnerships even beyond the current pandemic. It behooves us all to develop mechanisms to harness academic-public sector engagement so that we can be even more agile in uplifting these vital partnerships into the future.

## Data Availability

eSchool + 2020–21 Tracking State and National School Reopening Plans: https://equityschoolplus.jhu.edu/reopening-policy-tracker/. eSchool + 2020–21 Teacher & School staff COVID-19 Vaccination Dashboard: https://equityschoolplus.jhu.edu/vaccinations-dashboard/. eSchool + 2021–22 National and Index School District Mask & COVID-19 Vaccine Policy Tracker: https://policies.equityschool.plus/school-plans/. eSchool + COVID-19 Global Education Recovery Tracker: https://www.covideducationrecovery.global/. Johns Hopkins Consortium for School-Based Health Solutions COVID-19 Health Education Modules: https://schoolhealth.jhu.edu/covid19_resources/modules/.

## Abbreviations

EUA: Emergency use authorization
FDA: Food & Drug Administration
GERT: Global education recovery tracker
K: Kindergarten
MACC: Multidisciplinary academic group on children and coronavirus
UNICEF: United Nations International Children’s Emergency Fund
US: United States
